# Correction: A long non-coding RNA is required for targeting centromeric protein A to the human centromere

**DOI:** 10.7554/eLife.07239

**Published:** 2015-03-04

**Authors:** Delphine Quénet, Yamini Dalal

Quénet D, Dalal Y. 2014. A long non-coding RNA is required for targeting centromeric protein A to the human centromere. *eLife* **3**:e03254. doi: 10.7554/eLife.03254. Published 12 August 2014

An error has been brought to our attention whereby there is duplication of two panels, within Figure 1A (top left panel) and a control panel inside Figure 3C (top left panel). Both images, which were inadvertently duplicated during figure preparation, display co-immuno-fluorescence of RNAPII^S2P^ and CENP-B performed on chromatin fibers extracted from eG1-synchronized HeLa cells.

In this correction, we replace the top left panel in Figure 3C with a different chromatin fiber acquired under exactly the same conditions. The conclusions are unaffected and we apologize for the original mistake.

The corrected Figure 3 is shown here:

**Figure 1:**
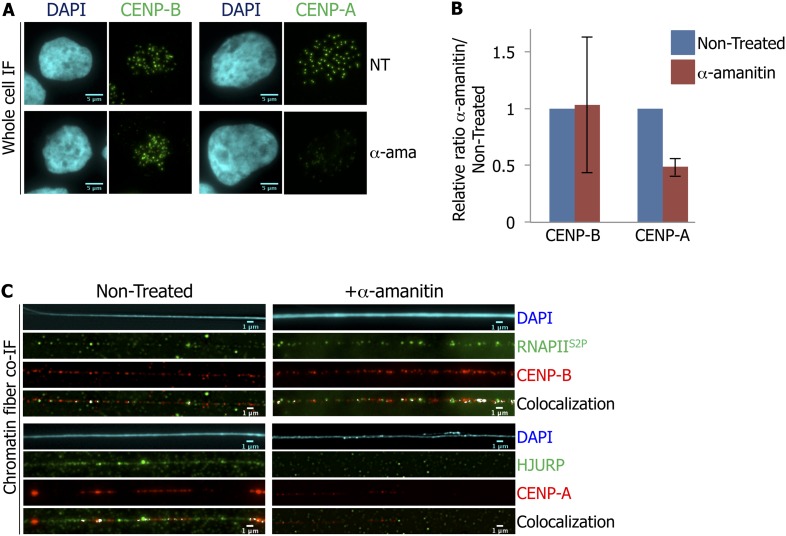

The originally published Figure 3 is also shown for reference:

**Figure 2:**
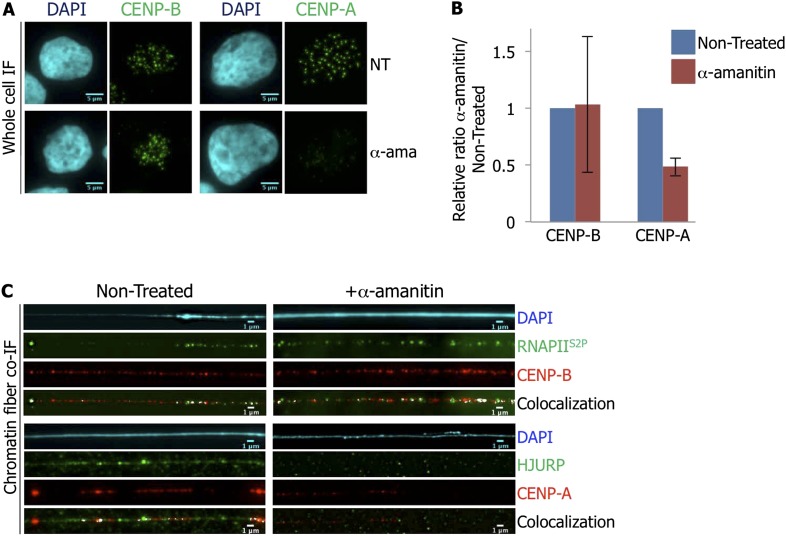

The article has been corrected accordingly.

