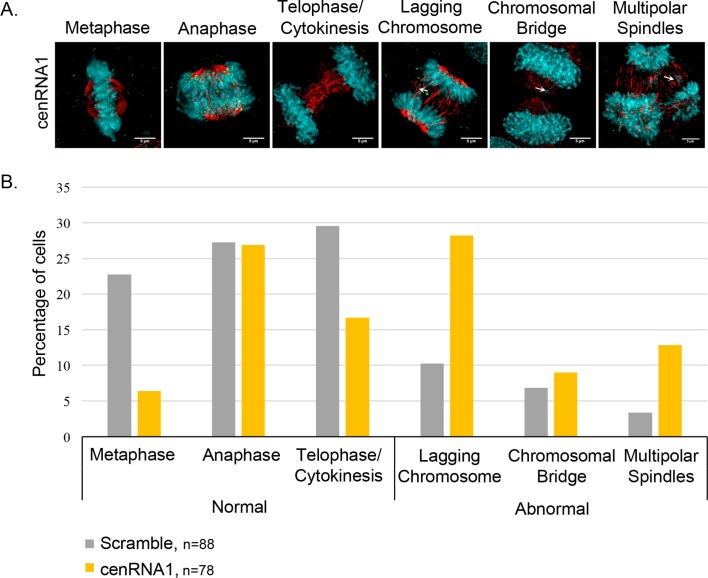# Correction: A long non-coding RNA is required for targeting centromeric protein A to the human centromere

**DOI:** 10.7554/eLife.41593

**Published:** 2018-11-01

**Authors:** Delphine Quénet, Yamini Dalal

Quénet D, Dalal Y. 2014. A long non-coding RNA is required for targeting centromeric protein A to the human centromere. *eLife* **3**:e03254. doi: 10.7554/eLife.03254.

Published 12, August 2014

We previously reported a centromeric long non-coding RNA called CenRNA#1, whose depletion by shRNA leads to mitotic defects (Quenet and Dalal, 2014). However, subsequent studies from our own laboratory (described herein) have yielded observations that are in conflict with our initial findings. While we stand by our original conclusion regarding the mitotic defects associated with the designed shRNA against the putative CenRNA#1, we have since discovered that the proposed sequence for cenRNA#1 arose from a technical artifact. In this correction, we report on the technical mistake made in our previous publication. We deeply regret our error, and apologize to the field.

To highlight differences between our original strategy and the revised one, we corrected the text as followed:

The article has been corrected accordingly, as detailed below:

“We next sought to purify, clone using a conventional TOPO T/A cloning strategy and sequence CENP-A-associated RNA “

The original version for reference was:

“We next sought to purify, clone, and sequence CENP-A-associated RNA”

Due to re-sequencing of the original cenRNA#1, we corrected the text to read:

“This sequencing approach was moderately successful, yielding one sequence of ~675 nucleotides (cenRNA#1, Figure 5 - figure supplement 1). This RNA sequence is unique and contains four semi-regular spaced 28 bp repeats with a weak homology (~52%) to the canonical CENP-B box (Supplementary file 2)”

The original version for reference was:

“This sequencing approach was moderately successful, yielding one sequence of ∼665 nucleotides (cenRNA#1, Figure 5—figure supplement 1). This RNA sequence is unique and contains four semi-regularly spaced 28-bp repeats with a weak homology to the canonical CENP-B box (data not shown)”

We replaced text describing the putative centromeric origin of CenRNA#1 with a description of the discovery of its adapter composition. The replacement text follows:

“Over the course of the subsequent two years after publication, we made additional attempts to map centromeric RNAs, turning to a high-throughput approach coupled to CENP-A and HJURP RIP-Seq. These data yielded ~435 centromeric-mapping RNA sequences (Quenet, et al, 2016), which, by RNA FISH localize to centromeres (unpublished). However, the initial cloned CenRNA#1 sequence did not map to this database of centromeric RNAs. This led us to re-sequence 6 clones of CenRNA#1, which led to the rescue of 12 N’s and 27 base calls changed in the 675bp sequence (Figure 1). We then performed an expanded sequence search across multiple sequence databases beyond the publicly available NCBI catalog, including the HeLa genome (after obtaining permission from the HeLa DGAP working group), as well as an industrial database for cloning vectors (Supplementary files 2, 3, 4). Much to our dismay, we discovered that cenRNA#1 arose largely from fusions of adapters used in the RNA cloning approach and described in Pfeffer et al. (Figure 2; (Pfeffer et al., 2005;). Based on this evidence, we no longer believe that the original sequence provided for putative cenRNA#1 represents a human centromeric transcript.”

The removed text for reference was:

“Since most human centromeres remain intractable to complete sequencing (Lander et al., 2001), this outcome is not entirely surprising. We were unable to detect cenRNA#1 directly on Northern blots, presumably due to its low abundance or due to its instability. Therefore, to directly reveal the genomic origin of the sequenced RNA, we converted it to a DNA FISH probe (cenRNA#1DNA). We next either performed control IF/FISH using the Xist locus (XistDNA, on the q-arm of the X-chromosome) and CENP-A, IF/FISH with cenRNA#1DNA and CENP-A, or IF/double-FISH with CENP-A, cenRNA#1DNA and centromeric α-satellite probes, on chromatin fibers. As expected, control IF/FISH between XistDNA and CENP-A yielded no appreciable signal (Figure 5A). In contrast, about half the CENP-A, or centromeric α-satellite stained fibers, contain the cenRNA#1DNA signal (Figure 5B, left vs right, quantification below panel). This result is consistent with the fact that centromeric α-satellite DNA sequences are not strictly conserved across all human centromeres (Lander et al., 2001). Significantly, 100% of the cenRNA#1DNA was found associated with CENP-A (Figure 5B, top right panel), and with centromeric α-satellite DNA probe (Figure 5B, lower right panel), demonstrating robust association on centromeric chromatin fibers. Thus, these data (Figure 5) indicate that the sequenced lncRNA (Figure 5—figure supplement 1), which is associated with CENP-A at eG1 (Figure 4B), indeed has a centromeric origin.”

In light of the above, we revised our functional analysis and description of mitotic defects. The text now reads:

“In our initial version, we seek to assess the functional role of cenRNA#1 by an shRNA strategy to down-regulate specifically its expression (Figure 5 – figure supplement 1). The two shRNAs were generated from the putative sequence of cenRNA#1, against the 28bp repeat element sequence. Cells were transfected with control scrambled (shRNA^scram^) or shRNA^cenRNA#1^ constructs, and selected with puromycin Cells transfected with shRNA^scram^ displayed no changes in morphology and density, whereas cells treated with shRNA^cenRNA#1^ displayed significant loss of cell density (down by ~70%, relative to control), and presented aberrant morphology (Figure 5 – figure supplement 4A and 2B).

We decided to readdress this experiment. Based on the re-sequencing results, three base calls within each shRNA were changed, which removes their uniqueness within cenRNA#1 and changes the percent identity to 26/29 bases (Supplementary file 6). Neither shRNA had a significant match to the human genome or known transcripts. However, the best hit is to a lncRNA on chromosome 3 (15/29 bases), which is proximal to the centromere, but not in the pericentromere (Supplementary file 7). This proximity may potentially explain the positive IF/FISH signal observed in our initial manuscript of CENP-A with cenRNA#1DNA probe (Figure 5). An independent reproduction of the down-regulation of cenRNA#1 by shRNA approach yielded the same chromosome defect as before (M. Bui, data not shown). To re-test whether some fraction of cenRNA#1 matching sequence plays a role in chromosomal integrity, we next designed a locked nucleic acid antisense oligonucleotide (LNA ASO) and analyzed mitosis integrity. Indeed, LNA ASO targeting cenRNA#1 led to modestly increased rates of lagging chromosomes and cells with multi-polar spindles, when compared to either un-transfected, mock transfected, or scrambled transfected cells (28% and 13% compared to 10% and 3% for scrambled control; (Figure 5 – figure supplement 5). This result may suggest a potential function for some fraction of this non-centromeric sequence on chromosome segregation and mitotic integrity, but which is not connected to the main findings of our original manuscript.”

The original text for reference was:

“Next, we assessed the functional role of cenRNA#1 by an shRNA strategy to down-regulate specifically its expression (Figure 5—figure supplement 1). Cells were transfected with control scrambled (shRNA^scram^) or shRNA^cenRNA#1^ constructs and selected with puromycin. Cells transfected with shRNA^scram^ displayed no changes in morphology and density, whereas cells treated with shRNA^cenRNA#1^ displayed significant loss of cell density (down by ∼70%, relative to control) and presented aberrant morphology (Figure 5—figure supplement 2A,B). Altogether these data suggest that cenRNA#1 transcript significantly affects cell integrity.”

The following Materials and Methods text has been modified:

“Supplementary file 8 lists all antibodies used for each experiment”

The original text for reference was:

Supplementary file 2 lists all antibodies used for each experiment”

The following Materials and Methods text has been modified:

“The sequences of the radiolabeled probes are indicated in Supplementary file 10”

The original text for reference was:

“The sequences of the radiolabeled probes are indicated in Supplementary file 3”

The following new Materials and Methods text has been added:

LNA ASOs were designed and purchased by QIAGEN (previously Exiqon). Supplementary file 9 lists sequences of these LNA ASO sequence.

Down-regulation of cenRNA#1

Transfection of HeLa cells with LNA ASO were conducted as in (Bui et al., 2017). Briefly, cells were seeded 24 hours before transfection to allow no more than 75% confluency, and transfected using Lonza’s Amaxa Cell Line Nucleofector Kit R (Cat #VCA-1001) with Amaxa Biosystems Nucleofector II electroporation system using program O-005. After transfection, cells were grown on coverslips with fresh DMEM medium.

Immuno-fluorescence of mitotic cells

The day following LNA ASO transfection, cells were synchronized with a double thymidine block and released for 10.5 hours on the third day, to enrich for anaphase-cytokinesis staged cells. IF experiments were performed as described previously (Bui et al., 2012). Cells were fixed with 4% paraformaldehyde in 1X PBS (#14190-144; Gibco by Life Technologies) for 15 min, permeabilized with 0.5% Triton X-100 in 1X PBS for 10 min, and blocked with 3% bovine serum albumin (BSA, #BP9706-100; Fisher Scientific) in 1X PBS. Coverslips were immuno-stained for CENP-C and α-tubulin for one hour each (Supplementary file 8). After three washes in 1X PBS, 0.1% Tween (#P7949-500ML; Sigma-Aldrich), cells were incubated with secondary antibody (goat anti-guinea pig or anti-mouse IgG (H+L) secondary antibodies, Alexa Fluor568 and Alexa Fluor488 conjugates (Thermo Fisher Scientific)) in 1X PBS for 1 hour at RT in the dark. Finally, cells were washed three times for 5 min at RT. Coverslips were mounted with anti-fade mounting medium Prolong Gold with DAPI.

Microscopy observation and analysis

IF slides were observed with a DeltaVision Elite RT microscopy imaging system (GE Healthcare) controlling an interline charge-coupled device camera (Coolsnap) mounted on an inverted microscope (IX-70; Olympus). Images were captured by using a 60X objective at 0.2μm z-sections and analyzed with Image J (1.50e; Java 1.6.0_20).”

The acknowledgements section was updated:

"We thank Drs. Shiv Grewal (LBMB, NCI), Thomas Misteli (LRBGE, NCI), Rachel O'Neill, Steven Henikoff, Gordon Hager, and members of our lab for thoughtful discussion and critical feedback;James McNally (LRBGE, NCI) and Kathy McKinnon (Vaccine Branch, NCI) for the access to microscopy facility and FACS core facility, respectively.YD and DQ are supported by the Intramural Research Program of the Center for Cancer Research at the National Cancer Institute/NIH. Dr. David Sturgill (staff bioinformatician, LRBGE, NCI) discovered the correct identity of cenRNA1 by performing the extensive analysis discussed in the correction, and Dr. Minh Bui (lab biologist, LRBGE, NCI) performed rigorous ASO/LNA analysis to understand why the cenRNA1 knockdown yielded a mitotic defect observed in the original manuscript." 

The original text was:

"We thank Drs. Shiv Grewal (LBMB, NCI), Thomas Misteli (LRBGE, NCI), Rachel O'Neill, Steven Henikoff, Gordon Hager, and members of our lab for thoughtful discussion and critical feedback;James McNally (LRBGE, NCI) and Kathy McKinnon (Vaccine Branch, NCI) for the access to microscopy facility and FACS core facility, respectively. YD and DQ are supported by the Intramural Research Program of the Center for Cancer Research at the National Cancer Institute/NIH."

The following supplementary files have been added:

Supplementary file 2: Alignment of cenRNA#1 28bp repeat to CENP-B box.

**Table:** 

Score: 500	Alignment
Sequence CENP-B: 1-17 Sequence 28bp: 1-27	CENP-B: CT– – –TCGTTGGAAA–CGGGA | | | | | | | | | | | 28bp seq: CTAAAT– –TT– – –AACCGCGA
Length of alignment: 21 bases	Percentage ID: 52.38

Supplementary file 3: Best alignment hits for cenRNA#1 regions without contiguous full-length adapter sequences.

**Table:** 

Method	Database	Results
BLAST Alignment	Human genome reference hg19	No significant alignment: Shorter contiguous hit (20 bp) than the longest contiguous hit in a random sequence of the same length and GC content (23 bp)
	Human transcript sequence	No significant alignment: With an average exon size of 123 bases (Scherer and Basso, 2008), unlike cenRNA#1 transcription from short exons separated by long introns
	extended higher-order repeats from chromosome X from (Miga et al., 2014)	No significant alignment
	hg38 reference assembly, incorporating several megabases of new centromeric assembly	No significant alignment
	α-satellite monomers and novel repeating centromeric elements from (Lacoste et al., 2014)	No significant alignment
	α-satellite monomers and novel repeating centromeric elements from (Henikoff et al., 2015)	No significant alignment
	VecScreen	Some similarities with vector sequence
	nr database	No significant alignment
	small RNAs	No significant alignment
	viral sequences	No significant alignment
	HeLa genome (Adey et al., 2013; Landry et al., 2013)	No significant alignment

Supplementary file 4: List of databases and sequences questioned to identify cenRNA#1 origin

**Table:** 

NCBI databases	-Human genome + transcript (Build 38) -Non-redundant nucleotide collection (nr/nt) -Expressed sequence tags (EST) -Genomic survey sequences (gss) -High-throughput genomic sequences (hgts) -Whole genome shotgun contigs (wgs) -Transcriptome shotgun assembly seqs (TSA)
SRA studies	-DRX000595: HeLa.Std.5000ng.1st DRP000372 • Unamplified Cap Analysis of Gene Expression on a single molecule sequencer (HeliScopeCAGE) -DRX001262: HeLa cells (without wild-type U2AF35 induction by doxycycline) DRP000527 • RNA sequencing of wild-type or mutant U2AF35 transduced HeLa cells -DRX002709: Chromatin immunoprecipitated DNA of CFIm68 in control HeLa cells DRP000897 • Illumina sequencing of CFIm68 binding regions in HeLa cells -DRX002710: Input DNA for CFIm68 ChIP of control HeLa cells DRP000897 • Illumina sequencing of CFIm68 binding regions in HeLa cells -DRX013196: HeLa PolII DRP001297 • Construction of Mate Pair Full-length cDNAs Libraries and Characterization of Transcriptional Start Sites and Termination Sites -ERX634046: CLIP-Seq of H. sapiens HeLa cells to investigate transcriptome-wide mapping of PTB / hnRNP I CLIP-Seq of H. sapiens HeLa cells to investigate transcriptome-wide mapping of PTB / hnRNP I -SRX749241: RnaSeq_HeLa_cell_RNaseRSRP049453 • Homo sapiens Transcriptome or Gene expression -SRX749316: RnaSeq_HeLa_cell_RibominusSRP049453 • Homo sapiens Transcriptome or Gene expression -ERX615573: Whole Genome Sequencing of human -SRX699196: whole genome sequence of HeLa cells: Sample JR6-UPL_ACTTGAStudy summary: SRP046745 • HeLa Genome Sequencing -SRX699188: whole genome sequence of HeLa cells: Sample JR5-QPCR_CGTACG SRP046745 • HeLa Genome Sequencing
dbGaP	-Epigenetic Profiling of Human Colorectal Cancer (phs000385.v1.p1) -HeLa Cell Genome Sequencing Studies (phs000640.v3.p1)
Specialized databases	-DASHR: database of small human noncoding RNAs (Leung et al., 2016) -deepBase v2.0 (Zheng et al., 2016) -EMBL Rfam database (Database of RNA family domains) -mIRbase -RNAcentral

Supplementary file 5: Best alignment hits for cenRNA#1 regions without contiguous full-length adapter sequences

**Table:** 

ID	Sequence	Best hits in Pfeffer’s article (Pfeffer et al., 2005)	Best hit in human genome
Region 1: 21bp	AGCCAACGGAATTCCTTTGGC	p2 (16/16 100%) a2 (11/11 100%)	chr10:3723839-3723855 (17/17 100%)
Region 2: 23bp	CGCGAATTCCAGCTAGTCCAGCC	p2 (18/18, 100%) a1 (12/12 100%)	chr11:96647943-96647960 (18/18, 100%) chr7:131446816-131446832 (17/17, 100%) chrX:24111144-24111160 (17/17 100%)
Region 3: 64bp	TCAGCCAACGGAATTCCTCACTAACCGCGAATTCCAGCTAGTCAGCCAACGGAATTCCAGCTAGT	p2 (23/23 100%) a2 (15/15 100%) multiple short hits	chr2:22737705- 22737730 (23/26, 88.5%)

Supplementary file 6: Changes to cenRNA#1 shRNA sequence.

**Table:** 

shRNA		
shRNA-A	Previous sequence	CAAGCTAGTCAGCCAA**T**G**C**AATTCCTCA**T**
	New sequence	CAAGCTAGTCAGCCAA**C**G**G**AATTCCTCA**C**
shRNA-B	Previous sequence	**T**GCTAG**A**CAGCCAA**T**G**C**AATTCCTCA**T**TA
	New sequence	**A**GCTAG**T**CAGCCAA**C**G**G**AATTCCTCA**C**TA

Supplementary file 7: Alignment of cenRNA#1 shRNA sequence.

**Table:** 

Target	Alignment statistics
long intergenic non-protein coding RNA 971 (LINC00971)	Identities: 15/15 Query: ^7^ AGTCAGCCAACGGAA^21^ | | | | | | | | | | | | | | | Subject: ^722^ AGTCAGCCAACGGAA^736^
Sequence ID: NR_033860.1	Length: 7319 (chr3: 84638405-84869575)

Supplementary file 9: List of LNA ASO sequences and LNA probes.

**Table:** 

Target	Sequence (5’ to 3’)	Method
Scrambled	[*C]*A*CTAGCTGGAATTCCGT*G*G[*G]	Down-regulation
cenRNA#1	[*G]*A*CTAGCTGGAATTCCGT*G*G[*C]	Down-regulation

*denotes LNA modification

The following supplementary files have been re-numbered:

Supplementary file 2: List of antibodies used in this study.”

Has been re-numbered

Supplementary file 8: List of antibodies used in this study.”

Supplementary file 3: List of primer sequences used in this study.

Has been re-numbered

Supplementary file 10: List of primer sequences used in this study.”

The following changes have been made to Figure 5 – figure supplements:

Figure 5- figure supplement 2

has been re-numbered to

Figure 5- figure supplement 4

Additional Figure 5 – figure supplements have been added, as follow:

**Figure 5 – figure supplement 2**. Alignment of the original cenRNA1 with new sequencing results. Adjustment was performed when similar uncalled bases (12 N’s) or called bases (27) were found in the six clones (~6% of total sequence changed).

**Figure 1:**
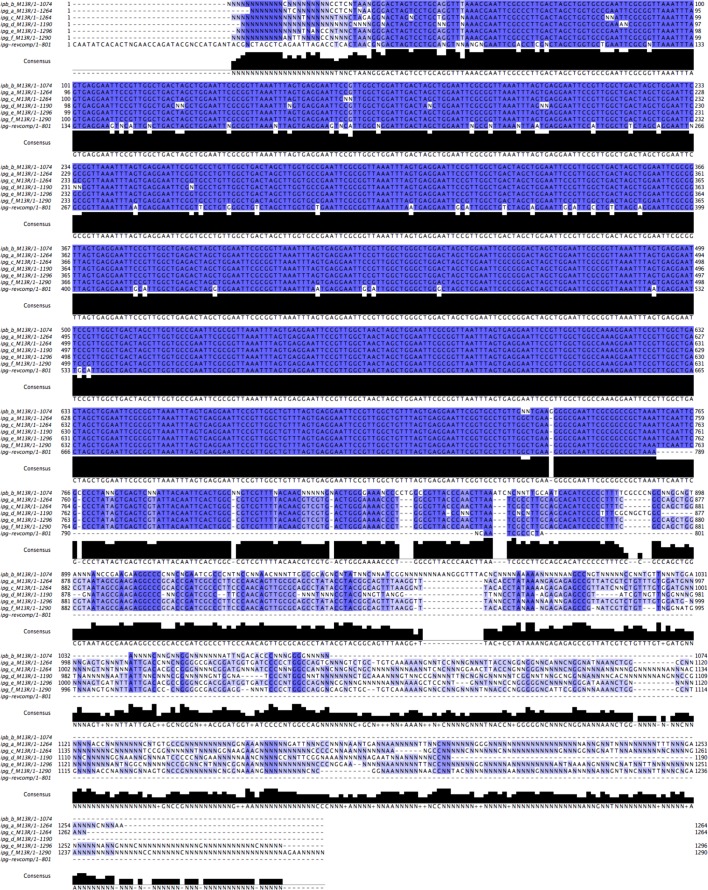


**Figure 5 – figure supplement 3**. Map of re-sequenced cenRNA#1. Sequence alignments revealed that cenRNA#1 is principally composed of adapters used for cloning step described in (Pfeffer et al., 2005). Only full-length, 100% identical locations of Pfeffer et al. sequences are indicated (except at sequence ends).

**Figure 2:**
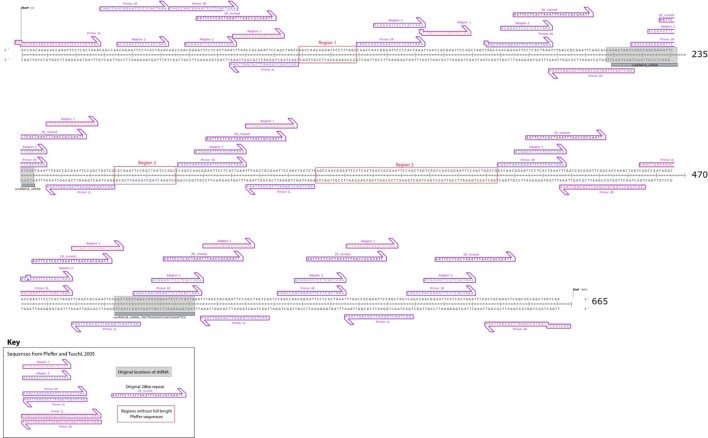


**Figure 5 – figure supplement 5**. The down-regulation of cenRNA#1 leads to chromosome defects. (A) Transfected HeLa cells with cenRNA#1 was synchronized at mitosis - early G1. Then, cells were stained for CENP-C (marker of centromeres) and α-tubulin (marker of mitotic spindle). Mitoses were categorized for their chromosome defects (lagging chromosomes, chromosomal bridges or multi-polar spindles-MPS). Scale bar: 5 μm. (B) Mitotic defects were counted and percentages were diagrammed.

**Figure 3:**