# Supplementary material for: A long non-coding RNA is required for targeting centromeric protein A to the human centromere
Source: eLife. 2014 Aug 12;3:e26016. doi: 10.7554/eLife.03254 (PMC4145801; doi:10.7554/eLife.03254)
Supplement: Supplementary file 1. — eG1 synchronized cells were treated 2 hr with or without α-amanitin and stained for centromeric protein CENP-A or CENP-B. After image acquisition, immunofluorescent signals were quantified using ImageJ. [file elife-03254-supp1.docx]

**Supplementary File 1: RNA Polymerase II inhibition results in CENP-A loss at centromere at early G1.** eG1 synchronized cells were treated 2 hrs with or without α-amanitin, and stained for centromeric protein CENP-A or CENP-B. After image acquisition, immuno-fluorescent signals were quantified using ImageJ.

| Staining | CENP-B | | CENP-A | |
| --- | --- | --- | --- | --- |
| Treatment | Non-Treated | α-amanitin | Non-Treated | α-amanitin |
| Relative ratio | 1.000 | 1.032 | 1.000 | 0.488 |
| Standard deviation | 0.000 | 0.596 | 0.000 | 0.077 |
| Number of cells measured | 73 | 76 | 91 | 55 |
| *p*-value | 0.9309 | | 0.0003 | |
