# Supplementary material for: A long non-coding RNA is required for targeting centromeric protein A to the human centromere
Source: eLife. 2014 Aug 12;3:e26016. doi: 10.7554/eLife.03254 (PMC4145801; doi:10.7554/eLife.03254)
Supplement: Supplementary file 2. [file elife-03254-supp2.docx]

**Supplementary file 2: Alignment of cenRNA#1 28bp repeat to CENP-B box.**

| Score: 500 | Alignment |
| --- | --- |
| Sequence CENP-B: 1-17  Sequence 28bp: 1-27 | CENP-B: CT– – –TCGTTGGAAA–CGGGA  \| \| \| \| \| \| \| \| \| \| \|  28bp seq: CTAAAT– –TT– – –AACCGCGA |
| Length of alignment: 21 bases | Percentage ID: 52.38 |
