# Supplementary material for: A long non-coding RNA is required for targeting centromeric protein A to the human centromere
Source: eLife. 2014 Aug 12;3:e26016. doi: 10.7554/eLife.03254 (PMC4145801; doi:10.7554/eLife.03254)
Supplement: Supplementary file 3. [file elife-03254-supp3.docx]

**Supplementary file 3: Best alignment hits for cenRNA#1 regions without contiguous full-length adapter sequences.**

| **Method** | **Database** | **Results** |
| --- | --- | --- |
| BLAST Alignment | Human genome reference hg19 | No significant alignment: Shorter contiguous hit (20 bp) than the longest contiguous hit in a random sequence of the same length and GC content (23 bp) |
|  | Human transcript sequence | No significant alignment: With an average exon size of 123 bases (Scherer and Basso, 2008), unlike cenRNA#1 transcription from short exons separated by long introns |
|  | extended higher-order repeats from chromosome X from (Miga et al., 2014) | No significant alignment |
|  | hg38 reference assembly, incorporating several megabases of new centromeric assembly | No significant alignment |
|  | α-satellite monomers and novel repeating centromeric elements from (Lacoste et al., 2014) | No significant alignment |
|  | α-satellite monomers and novel repeating centromeric elements from (Henikoff et al., 2015) | No significant alignment |
|  | VecScreen | Some similarities with vector sequence |
|  | nr database | No significant alignment |
|  | small RNAs | No significant alignment |
|  | viral sequences | No significant alignment |
|  | HeLa genome (Adey et al., 2013; Landry et al., 2013) | No significant alignment |
