# Supplementary material for: A long non-coding RNA is required for targeting centromeric protein A to the human centromere
Source: eLife. 2014 Aug 12;3:e26016. doi: 10.7554/eLife.03254 (PMC4145801; doi:10.7554/eLife.03254)
Supplement: Supplementary file 4. [file elife-03254-supp4.docx]

**Supplementary file 4: List of databases and sequences questioned to identify cenRNA#1 origin**

| **NCBI databases** | -Human genome + transcript (Build 38)  -Non-redundant nucleotide collection (nr/nt)  -Expressed sequence tags (EST)  -Genomic survey sequences (gss)  -High-throughput genomic sequences (hgts)  -Whole genome shotgun contigs (wgs)  -Transcriptome shotgun assembly seqs (TSA) |
| --- | --- |
| **SRA studies** | -DRX000595: HeLa.Std.5000ng.1st DRP000372 • Unamplified Cap Analysis of Gene Expression on a single molecule sequencer (HeliScopeCAGE)  -DRX001262: HeLa cells (without wild-type U2AF35 induction by doxycycline) DRP000527 • RNA sequencing of wild-type or mutant U2AF35 transduced HeLa cells  -DRX002709: Chromatin immunoprecipitated DNA of CFIm68 in control HeLa cells DRP000897 • Illumina sequencing of CFIm68 binding regions in HeLa cells  -DRX002710: Input DNA for CFIm68 ChIP of control HeLa cells DRP000897 • Illumina sequencing of CFIm68 binding regions in HeLa cells  -DRX013196: HeLa PolII DRP001297 • Construction of Mate Pair Full-length cDNAs Libraries and Characterization of Transcriptional Start Sites and Termination Sites  -ERX634046: CLIP-Seq of H. sapiens HeLa cells to investigate transcriptome-wide mapping of PTB / hnRNP I CLIP-Seq of H. sapiens HeLa cells to investigate transcriptome-wide mapping of PTB / hnRNP I  -SRX749241: RnaSeq_HeLa_cell_RNaseRSRP049453 • Homo sapiens Transcriptome or Gene expression  -SRX749316: RnaSeq_HeLa_cell_RibominusSRP049453 • Homo sapiens Transcriptome or Gene expression  -ERX615573: Whole Genome Sequencing of human  -SRX699196: whole genome sequence of HeLa cells: Sample JR6-UPL_ACTTGAStudy summary: SRP046745 • HeLa Genome Sequencing  -SRX699188: whole genome sequence of HeLa cells: Sample JR5-QPCR_CGTACG SRP046745 • HeLa Genome Sequencing |
| **dbGaP** | -Epigenetic Profiling of Human Colorectal Cancer (phs000385.v1.p1)  -HeLa Cell Genome Sequencing Studies (phs000640.v3.p1) |
| **Specialized databases** | -DASHR: database of small human noncoding RNAs (Leung et al., 2016)  -deepBase v2.0 (Zheng et al., 2016)  -EMBL Rfam database (Database of RNA family domains)  -mIRbase  -RNAcentral |
