# Supplementary material for: A long non-coding RNA is required for targeting centromeric protein A to the human centromere
Source: eLife. 2014 Aug 12;3:e26016. doi: 10.7554/eLife.03254 (PMC4145801; doi:10.7554/eLife.03254)
Supplement: Supplementary file 5. [file elife-03254-supp5.docx]

**Supplementary file 5: Best alignment hits for cenRNA#1 regions without contiguous full-length adapter sequences**

| **ID** | **Sequence** | **Best hits in Pfeffer’s article** **(Pfeffer et al., 2005)** | **Best hit in human genome** |
| --- | --- | --- | --- |
| Region 1: 21bp | AGCCAACGGAATTCCTTTGGC | p2 (16/16 100%)  a2 (11/11 100%) | chr10:3723839-3723855  (17/17 100%) |
| Region 2: 23bp | CGCGAATTCCAGCTAGTCCAGCC | p2 (18/18, 100%)  a1 (12/12 100%) | chr11:96647943-96647960  (18/18, 100%)  chr7:131446816-131446832  (17/17, 100%)  chrX:24111144-24111160  (17/17 100%) |
| Region 3: 64bp | TCAGCCAACGGAATTCCTCACTAACCGCGAATTCCAGCTAGTCAGCCAACGGAATTCCAGCTAGT | p2 (23/23 100%)  a2 (15/15 100%)  multiple short hits | chr2:22737705- 22737730  (23/26, 88.5%) |
