# Supplementary material for: A long non-coding RNA is required for targeting centromeric protein A to the human centromere
Source: eLife. 2014 Aug 12;3:e26016. doi: 10.7554/eLife.03254 (PMC4145801; doi:10.7554/eLife.03254)
Supplement: Supplementary file 6. [file elife-03254-supp6.docx]

**Supplementary file 6: Changes to cenRNA#1 shRNA sequence.**

| shRNA |  | |
| --- | --- | --- |
| shRNA-A | Previous sequence | CAAGCTAGTCAGCCAA**T**G**C**AATTCCTCA**T** |
|  | New sequence | CAAGCTAGTCAGCCAA**C**G**G**AATTCCTCA**C** |
| shRNA-B | Previous sequence | **T**GCTAG**A**CAGCCAA**T**G**C**AATTCCTCA**T**TA |
|  | New sequence | **A**GCTAG**T**CAGCCAA**C**G**G**AATTCCTCA**C**TA |
