# Supplementary material for: A long non-coding RNA is required for targeting centromeric protein A to the human centromere
Source: eLife. 2014 Aug 12;3:e26016. doi: 10.7554/eLife.03254 (PMC4145801; doi:10.7554/eLife.03254)
Supplement: Supplementary file 7. [file elife-03254-supp7.docx]

**Supplementary file 7: Alignment of cenRNA#1 shRNA sequence.**

| Target | Alignment statistics |
| --- | --- |
| long intergenic non-protein coding RNA 971 (LINC00971) | Identities: 15/15  Query: ^7^ AGTCAGCCAACGGAA^21^  \| \| \| \| \| \| \| \| \| \| \| \| \| \| \|  Subject: ^722^ AGTCAGCCAACGGAA^736^ |
| Sequence ID: NR_033860.1 | Length: 7319 (chr3: 84638405-84869575) |
