# Supplementary material for: A long non-coding RNA is required for targeting centromeric protein A to the human centromere
Source: eLife. 2014 Aug 12;3:e26016. doi: 10.7554/eLife.03254 (PMC4145801; doi:10.7554/eLife.03254)
Supplement: Supplementary file 8. [file elife-03254-supp8.docx]

**Supplementary File 8: List of antibodies used in this study**.

| Name | Origin | Immuno-precipitation | Dilution for Western Blot | Dilution for immuno-fluorescence |
| --- | --- | --- | --- | --- |
| α-tubulin | DM1A, Santa Cruz |  |  | 1:100 |
| β-actin | ACTBD11B7, Santa Cruz |  |  | 1:100 |
| CENP-A | custom |  | 1:1000 |  |
| CENP-A | 3-19, Abcam |  |  | 1:40000 (cell), 1:200 (ChF) |
| CENP-A | H50, Santa Cruz | 3 μg |  |  |
| CENP-B | ab25734 ,Abcam |  | 1:1000 | 1:400 (cell), 1:50 (ChF) |
| CENP-C | Ab33034, Abcam | 3 μg | 1:1000 | 1:400 |
| HJURP | Bethyl | 5 μg | 1:2000 |  |
| HJURP | H277, Santa Cruz |  | 1:1000 | 1:400 (cell), 1:50 (ChF) |
| TBP | Ab63766, Abcam |  | 1:2000 | 1:500 |
| Activated RNA Polymerase II (RNAPII^S2P^) | H5, Covance | 1:100 | 1:500 | 1:50 |
| RNA Polymerase II | CTD4H8, Santa Cruz |  | 1:1000 |  |

ChF: chromatin fiber
