# Supplementary material for: A long non-coding RNA is required for targeting centromeric protein A to the human centromere
Source: eLife. 2014 Aug 12;3:e26016. doi: 10.7554/eLife.03254 (PMC4145801; doi:10.7554/eLife.03254)
Supplement: Supplementary file 9. [file elife-03254-supp9.docx]

**Supplementary file 9: List of LNA ASO sequences and LNA probes.**

| Target | Sequence (5’ to 3’) | Method |
| --- | --- | --- |
| Scrambled | [*C]*A*CTAGCTGGAATTCCGT*G*G[*G] | Down-regulation |
| cenRNA#1 | [*G]*A*CTAGCTGGAATTCCGT*G*G[*C] | Down-regulation |

*denotes LNA modification
