# Supplementary material for: A long non-coding RNA is required for targeting centromeric protein A to the human centromere
Source: eLife. 2014 Aug 12;3:e26016. doi: 10.7554/eLife.03254 (PMC4145801; doi:10.7554/eLife.03254)
Supplement: Supplementary file 10. [file elife-03254-supp10.docx]

**Supplementary File 10: List of primer sequences used in this study.**

| Oligonucleotides | Sequence |
| --- | --- |
| CenRNA#1 Forward* | 5’-GCTAGTCAGCCAATGCAATTC-3’ |
| CenRNA#1 Reverse* | 5’-TGAGGAATTCGGTTCCTGTG-3’ |
| Centromeric α-satellite Forward[35]* | 5’-CATCACAAAGAAGTTTCTGAGAATGCTTC-3’ |
| Centromeric α-satellite Reverse [35]* | 5’-TGCATTCAACTCACAGAGTTGAACCTTCC-3’ |
| β-actin Forward | 5’-AACTGGAACGGTGAAGGTGACAGC-3’ |
| β-actin Reverse | 5’-TGGCTTTTAGGATGGCAAGGGAC-3’ |
| GAPDH Forward | 5’-GCGGTTCCGCACATCCCGGTAT-3’ |
| GAPDH Reverse | 5’-CCCCACGTCGCAGCTTGCCTA-3’ |
| 18S Forward | 5’-CGACGACCCATTCGAACGTCT-3’ |
| 18S Reverse | 5’-CTCTCCGGAACTGAACCCTGA-3’ |

*These primers were also used for RNA detection by Northern blot, after their tagging by DNA 5’ end labeling.
